# Nano-fibre Integrated Microcapsules: A Nano-in-Micro Platform for 3D Cell Culture

**DOI:** 10.1038/s41598-019-50380-0

**Published:** 2019-09-27

**Authors:** Shalil Khanal, Shanta R. Bhattarai, Jagannathan Sankar, Ramji K. Bhandari, Jeffrey M. Macdonald, Narayan Bhattarai

**Affiliations:** 1 Department of Applied Science and Technology, North Carolina A&T State University, Greensboro, NC USA; 2 Department of Chemistry, North Carolina A&T State University, Greensboro, NC USA; 3 Department of Biology, North Carolina A&T State University, Greensboro, NC USA; 4 Department of Biology, University of North Carolina Greensboro, Greensboro, NC USA; 5 Department of Mechanical Engineering, North Carolina A&T State University, Greensboro, NC USA; 6 Department of Biomedical Engineering, University of North Carolina, Chapel Hill, NC USA; 7 Department of Chemical, Biological, and Bioengineering, North Carolina A&T State University, Greensboro, NC USA

**Keywords:** Biomaterials - cells, Synthesis and processing, Nanocomposites

## Abstract

Nano-in-micro (NIM) system is a promising approach to enhance the performance of devices for a wide range of applications in disease treatment and tissue regeneration. In this study, polymeric nanofibre-integrated alginate (PNA) hydrogel microcapsules were designed using NIM technology. Various ratios of cryo-ground poly (lactide-co-glycolide) (PLGA) nanofibres (CPN) were incorporated into PNA hydrogel microcapsule. Electrostatic encapsulation method was used to incorporate living cells into the PNA microcapsules (~500 µm diameter). Human liver carcinoma cells, HepG2, were encapsulated into the microcapsules and their physio-chemical properties were studied. Morphology, stability, and chemical composition of the PNA microcapsules were analysed by light microscopy, fluorescent microscopy, scanning electron microscopy (SEM), Fourier-Transform Infrared spectroscopy (FTIR), and thermogravimetric analysis (TGA). The incorporation of CPN caused no significant changes in the morphology, size, and chemical structure of PNA microcapsules in cell culture media. Among four PNA microcapsule products (PNA-0, PNA-10, PNA-30, and PNA-50 with size 489 ± 31 µm, 480 ± 40 µm, 473 ± 51 µm and 464 ± 35 µm, respectively), PNA-10 showed overall suitability for HepG2 growth with high cellular metabolic activity, indicating that the 3D PNA-10 microcapsule could be suitable to maintain better vitality and liver-specific metabolic functions. Overall, this novel design of PNA microcapsule and the one-step method of cell encapsulation can be a versatile 3D NIM system for spontaneous generation of organoids with *in vivo* like tissue architectures, and the system can be useful for numerous biomedical applications, especially for liver tissue engineering, cell preservation, and drug toxicity study.

## Introduction

There is an unmet need in the field of tissue engineering and drug therapy to overcome several scientific and technical challenges, including the inability to precisely control the spatial and temporal features of the cellular microenvironment, the lack of materials with desired functional properties, the requirement for large sample volumes, and low throughput^1^. Cells grown in 2D flat surfaces do not fully reflect the essential physiology of *in vivo* microenvironments^2^. Prolonged cell culture in 2D systems modifies the tissue-specific architecture (e.g. forced polarity, flattened cell shape, etc.), mechanical/biochemical signals and cell-to-cell communication, and eventually the response from 2D test system deviates from *in vivo* response^3^. To overcome these limitations and to better mimic *in vivo* conditions, different synthetic 3D cell culture platforms have been created using various methods: hanging‐drop^4^, forced‐floating^5^, matrices scaffolds^6^, and agitation-based approaches^7^. In native stage of living body, almost all the cells in tissues reside in a complex fibrous meshwork known as extracellular matrix (ECM). The remodeling of ECM is a key structural and biochemical support that accounts the cellular properties. Several recent studies have demonstrated that changing the architecture of synthetic ECM around cells could enhance retention of tissue-specific functions. A synthetic, engineered ECM in 3D systems can significantly impact cell proliferation, differentiation, and cell survival to reproduce tissue-drive component in *in vitro*^2,3^.

Encapsulation of tissue-specific cells in microspheres is one of the promising 3D techniques used in tissue engineering and cell therapy^8–10^, cell storage and preservation^11^, and for development of *in vitro* platforms for drug discovery and toxicity screening^12,13^. This technique refers to immobilization of cells within a semipermeable hydrogel that allows bi-directional diffusion of nutrients, oxygen, wastes, and secretion of biomolecules. In cell therapy, the semi-permeable hydrogel avoids the foreign invaders, such as immune cells and antibodies which can destroy encapsulated cells^14,15^. In addition, the hydrogel microenvironment has other advantages particularly the ease of handling of cells in a highly hydrated environment that mimic the natural ECM in tissues^2,14–16^. Different extrusion methods have been used for cell encapsulation including electrostatic^17^, coaxial airflow^18^, vibrational nozzle^19^ and jet cutting^20^.

Two main categories of hydrogels used extensively in cell encapsulation are: synthetic polymer-based hydrogels, such as poly(ethylene glycol) (PEG), 2-hydroxyethyl methacrylate (HEMA), poly(vinyl alcohol) (PVA), polyvinylpyrrolidone (PVP), and PLGA-co-PEG^21–24^, and natural polymer-based hydrogel such as alginate, chitosan, collagen, gelatin, hyaluronic acid^25–27^. Although synthetic hydrogels have greater control over gelation time, macroscopic structure, and degradation kinetics, natural polymer-based hydrogels retain biological cues to guide cell and tissue growth^16,28^. Currently, a variety of hybrid hydrogels are developed to overcome the inherent limitation of both natural and synthetic hydrogels^29,30^. Designing of hybrid hydrogels by incorporating micro- and nanoscale features of both natural and synthetic polymers are emerging tools in tissue engineering to create biomimetic environments within the 3D system that enhances several cellular functions with high temporal and spatial resolution^31–33^.

Alginate has been used extensively for 3D cell encapsulation because of its proven biocompatibility, relatively easy to prepare at physiological conditions in the presence of divalent cations, and easy to sterilize and storage^34,35^. However, alginate has poor biological properties in terms of cell adhesion, migration, and viability^36,37^. In addition, alginate hydrogel does not degrade *in vivo*, but rather dissolves when the divalent cations from hydrogel are replaced by monovalent ions. Techniques, such as partial oxidation^38^ and blending alginate with cell adhesion oligopeptide^39^ or other polymers^40–47^, have been reported to overcome these limitations.

Here, we hypothesized that the integration of electrospun PLGA nanofibres into alginate hydrogel using NIM technology could enhance the limitation of alginate hydrogel microcapsule. The electrospun PLGA nanofibres have proven biomimetic properties that enhance cell-cell communications and cell-matrix interactions to regenerate tissues^48,49^. The PLGA has been used in numerous FDA approved devices. However, PLGA membranes alone cannot provide adequate immunoisolation for cells^50^. The large-sized alginate hydrogel microcapsules (>500 µm) can protect cells from the immune system but seriously decrease the perfusion of oxygen, nutrients and metabolic waste into the core of the microcapsule which can lead to cell death in long term culture^51^. Therefore, PLGA nanofibre-integrated alginate hydrogel microcapsule with controllable size can be a novel platform for cell encapsulation and to overcome the limitation of both alginate hydrogel microcapsules and electrospun nanofibres. Additionally, the incorporation of PLGA nanofibres could act as fillers in a composite hydrogel that reinforce the hydrogel network by filling the interstitial voids, and help to improve some desirable properties of the microcapsules such as density and rigidity for processing and handling^52^, and physical protection to the encapsulated cells through a densely packed environment^53^. Although PLGA nanofibres have been known to provide support to enhance cell-material and cell-cell interactions within the hydrogel sandwiches^54–60^, information regarding incorporation of electrospun PLGA nanofibres into alginate microcapsules, in particular, is not currently available.

In the present study, using the electrospinning technique followed by cryogenic grinding and electrostatic encapsulation methods, PNA hydrogel microcapsules were synthesized. Various analytical tools and imaging techniques were used to study the impact of CPN on the size and stability of PNA microcapsules. The corresponding PNA hydrogel microcapsule products were used to encapsulate HepG2 cells as a model 3D cell culture platform. The viability and growth of HepG2 cells, their cellular metabolic activity within the PNA microcapsules were studied. The newly designed PNA microcapsule, an ECM mimicking architecture with NIM integrated system, could impact future translational applications of the microcapsules for the development of human bioartificial liver (BAL) devices, and for surrogates of human toxicity testing devices.

## Results

### Production of nanofibre and CPN particles of PLGA

**Figure 1:**
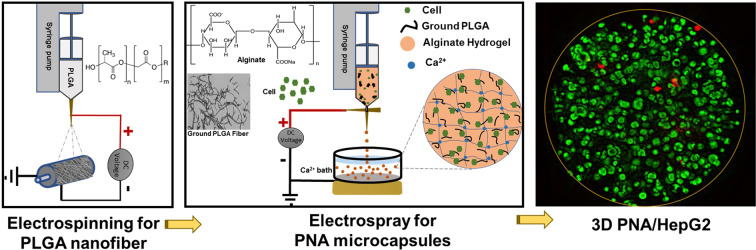
Schematic illustration for the production of PLGA nanofiber mesh, CPN particles, PNA microcapsules, and 3D PNA/HepG2 cell encapsulation. HepG2 cell encapsulated PNA microcapsules were obtained by using a custom-built cell encapsulation apparatus. Stained green and red colour in 3D PNA/HepG2 microcapsule (right side image) represent live and dead cells, respectively.

**Figure 2:**
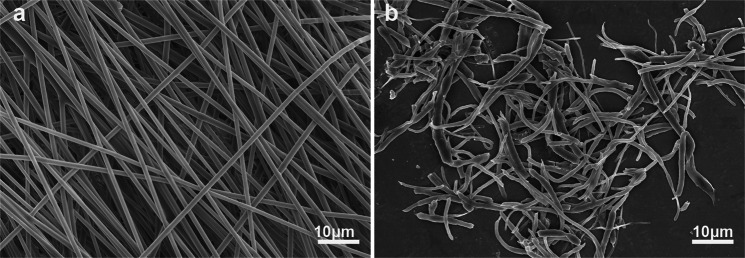
SEM images of electrospun nanofiber membrane (**a**) and CPN particles (**b**) of PLGA. (Nanofiber average diameter: 640 ± 121 nm and CPN particles size: 10–60 µm).

Figure 1 represents the step by step methodology for the PNA microcapsules preparation. Three different PLGA concentrations (15, 20, and 25 wt.%) were selected to examine nanofiber morphology and fibre diameters. 15% PLGA produced non-uniform fibre (calculated average diameter, 356 nm) with many beads and droplets embedded in the fibre networks. While smooth, randomly oriented (640 nm) fibres were obtained through 20% PLGA, and large diameter (969 nm) fibres were produced from 25% PLGA (Fig. S1). Fibre diameter was increased with increasing concentration of PLGA^48^. The fibres obtained from 20% PLGA (Fig. 2A) was found suitable to prepare CPN (Fig. 2B). The length of CPN particles was in the range of 10–60 µm with more fibrillated and chopped fibre morphology.

### Preparation and characterization of PNA hydrogel microcapsules

**Figure 3:**
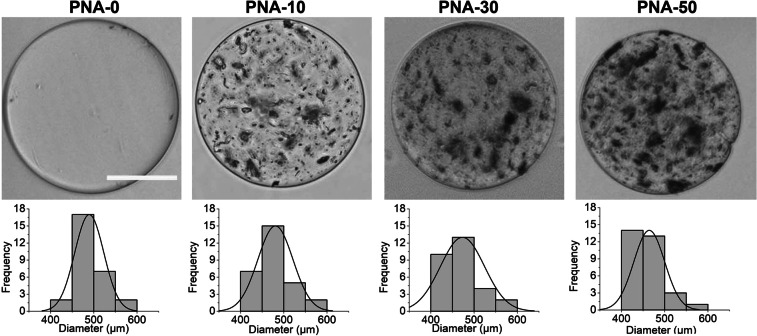
Optical images of representative single PNA microcapsule (top row) and corresponding size distribution (bottom row). The average diameter of PNA-0, PNA-10, PNA-30, and PNA-50 were 489 ± 31 µm, 480 ± 40 µm, 473 ± 51 µm and 464 ± 35 µm respectively. Scale bar = 200 µm.

Several electrospray parameters (e.g. voltage, distance to the collector, solution viscosity, and flow rate) were optimized to obtain reproducible PNA microcapsules with 10, 30, 50, 75 and 100 wt.% CPN particles. At 75 and 100% CPN particle concentrations, the spray solution was clogged into a needle which resulted in the random shape and poor quality of PNA microcapsules, hence were excluded for further experimental study. Figure 3 (top row) shows optical microscopic images of PNA microcapsules containing 0, 10, 30 and 50% (w/w) of CPN particles symbolled as PNA-0, PNA-10, PNA-30, and PNA-50, respectively. Microscopy images clearly indicated that PNA-0 microcapsules had opaque background and was changed into light dark background with increasing percentage of CPN particles. The external morphology of all PNA microcapsules was smooth and spherical. PNA-0 microcapsules were used as control microcapsules for comparative study. Figure 3 (bottom row) represents the size distribution of PNA microcapsules in the range of 400 to 600 µm. The average diameter was slightly decreased with increasing percentage of CPN particles and measured as 489 ± 31 µm, 480 ± 40 µm, 473 ± 51 µm and 464 ± 35 µm for PNA-0, PNA-10, PNA-30, and PNA-50, respectively.

The chemical structure and degradation behaviour of PNA microcapsules were analysed by FT-IR and TGA study, respectively (Fig. S2). The FT-IR spectra show that there were no changes in the position of absorption peaks for alginate hydrogel and CPN. CPN loaded microcapsules showed the peaks resulting from the superimposition of their separated components in the infrared spectra. All peaks found in CPN particles and alginate hydrogel alone were also present in the microcapsules containing CPN. PNA microcapsules stability was studied in cell culture media (DMEM with 10% FBS and 1% Pen- strip) by placing the PNA microcapsules on a rocker at 37 °C. There was no substantial change in morphology of all PNA microcapsules products up to 28 days (results not shown).

### HepG2 cells encapsulation in 3D PNA microcapsules

**Figure 4:**
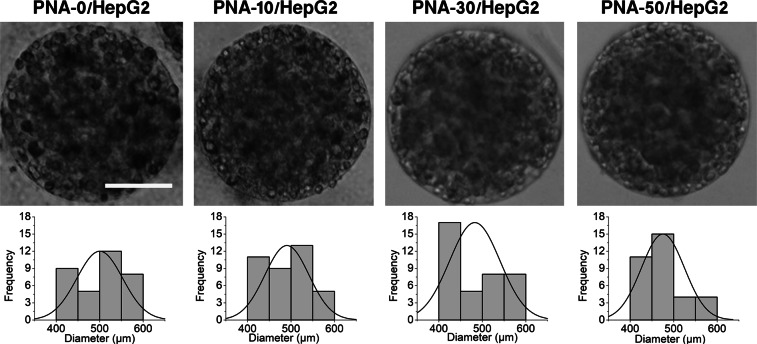
Optical images of representative single 3D PNA/HepG2 microcapsules (top row) and their respective size distribution (bottom row). The average diameter was measured as 500 ± 52 µm, 490 ± 50 µm, 482 ± 58 µm and 475 ± 49 µm for PNA-0, PNA-10, PNA-30, and PNA-50 microcapsules respectively. Scale bar = 200 µm.

The optical images of 3D PNA/HepG2 microcapsules produced in this study are shown in Fig. 4 (top row). 3D PNA-0/HepG2 microcapsules were used as control microcapsules for comparative study. The size of 3D PNA/HepG2 microcapsules was found in the range of 400–600 µm with spherical morphology and porous nature. The respective size distribution of 3D PNA/HepG2 microcapsules is shown in Fig. 4 (bottom row). The size of 3D PNA/HepG2 microcapsules was slightly bigger than control microcapsules. Among 3D PNA/HepG2 microcapsules, the average diameter was decreased with an increasing percentage of CPN particles and measured as 500 ± 52 µm, 490 ± 50 µm, 482 ± 58 µm and 475 ± 49 µm for PNA-0, PNA-10, PNA-30, and PNA-50, respectively. All cells were well distributed throughout the microcapsules with almost no cells on the surface of 3D PNA microcapsules.

### Fluorescence imaging of 3D PNA/Hepg2 microcapsules

**Figure 5:**
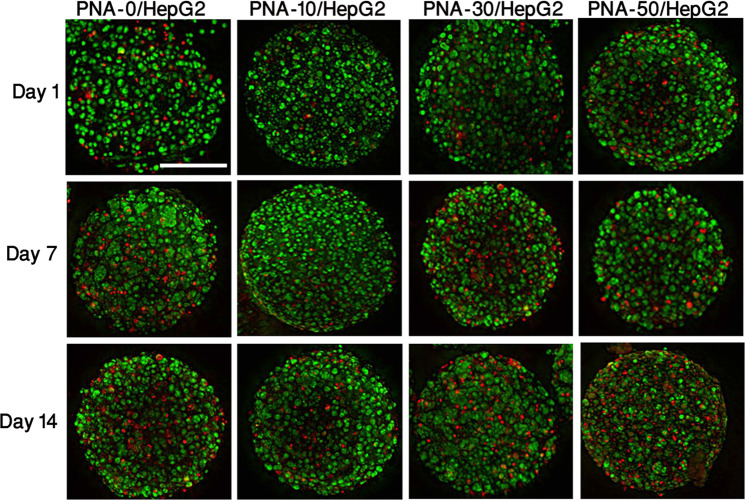
Fluorescent images of dye-labeled 3D PNA/HepG2 microcapsules at different time points. Green and red colour indicates live and dead cells, respectively, and were uniformly distributed in all compositions of the PNA microcapsules. Scale bar = 200 µm.

The 3D PNA/HepG2 microcapsules were stained with acridine orange propidium iodide (AOPI) dye, where the live and dead cells are stained as green and red, respectively (Fig. 5). The fluorescent images support the uniform cell distribution and morphological stability of 3D PNA/HepG2 microcapsules up to 14 days of culture. In all microcapsules the HepG2 grew with the formation of some clonal aggregates, and growth did not appear to be affected by the fibres. The external morphology of all the microcapsules was smooth and spherical with no visible difference in morphology over 14 days of culture observation. The spherical morphology and porous nature of the microcapsules were observed by the SEM (Fig. S4) and distribution of cells within the microcapsules was confirmed. However, the PNA-10/HepG2 microcapsules showed better viability (p < 0.05) compared to other compositions as they had a fewer number of dead cells (i.e. stained red) (Fig. S3).

### Cell viability of 3D PNA/HepG2 microcapsules

**Figure 6:**
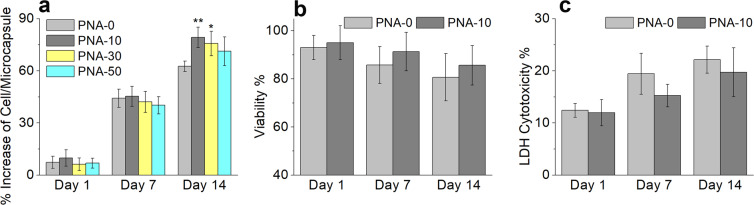
Cell growth and viability study. Cell growth per microsphere (**a**). Viability test using trypan blue assay (**b**) and LDH cytotoxicity (**c**) of encapsulated HepG2 in the PNA-0 and PNA-10 microcapsules at days 1, 7 and 14. Data represent the mean ± S.D. (n = 3). Tukey test, *p < 0.05, **p < 0.01, ***p < 0.001.

Viability of HepG2 cells in all four types of 3D PNA/HepG2 microcapsules i.e PNA-0, PNA-10, PNA-30, and PNA-50 was measured by trypan blue and LDH assay, and AOPI dye staining. The effect of CPN on the growth of encapsulated cells within the microcapsules was also studied by counting the number of cells per microcapsule at different time points. HepG2 cell growth was increased with increasing culture time (Fig. 6A). The cell growth in all CPN containing microcapsules (PNA-10, PNA-30, and PNA-50) was higher compared to control microcapsule (PNA-0). Significant effects of CPN on cell growth was noticed at day 14, and 10% CPN microcapsule showed the highest rate of cell growth (p < 0.01) compared to control microcapsule (Fig. 6A). Hence, subsequent studies mainly compared between PNA-0 and PNA-10 microcapsules. The viability of HepG2 cells at day 14 was 80 ± 9% and 85 ± 8% for PNA-0 and PNA-10 microcapsules, respectively. The PNA-10 microcapsules showed substantial higher viability at days 7 and 14 compared to PNA-0, (Fig. 6B). A similar pattern was observed in LDH levels (Fig. 6C) supporting the viability data (Fig. 6B).

### Metabolic activity of 3D PNA/HepG2 microcapsules

**Figure 7:**
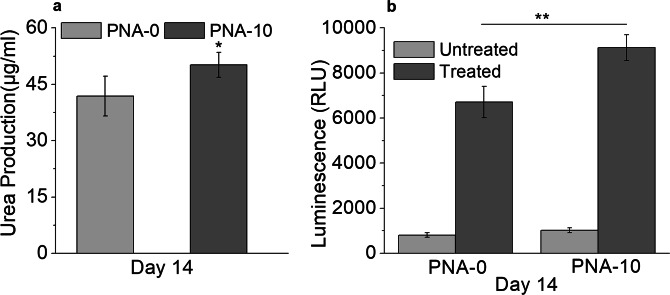
Metabolic and enzyme activity biomarkers: (**a**) Urea production, (**b**) CYP 1A1 activity by 3D PNA-0/HepG2 and 3D PNA-10/HepG2 microcapsules at day 14. 3D PNA-10/HepG2 encapsulates showed significantly higher urea production and greater activity for CYP 1A1 enzyme. Data represent the mean ± S.D (n = 3). Tukey test, *p < 0.05, **p < 0.01.

The level of urea production of 3D PNA/HepG2 microcapsules at day 14 is shown in Fig. 7A and found significantly (p < 0.05) higher in PNA-10 compared to PNA-0 microcapsules. In PNA-0/HepG2 microcapsules, the urea production level was decreased over time and found lower at day 14 compared to day 1 (data not shown), suggesting that ammonia detoxification may not be possible in 3D PNA-0/HepG2 encapsulates for long term culture. CYP1A1 enzyme activity was determined using luminescent CYP450-specific substrate after induction with omeprazole. The induced enzyme activity was increased, approximately 9-folds in PNA-10 compared to that of untreated microcapsules, Fig. 7B. These results indicate healthy maintenance of HepG2 cells within the PNA microcapsules. The fibrous structures in PNA microcapsules can allow encapsulated cells to grow well in terms of increasing cell population as well as enhancing cellular functions^60^.

### 3D PNA-10/primary hepatocytes microcapsules

**Figure 8:**
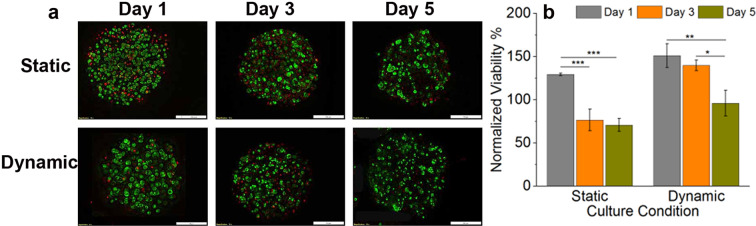
Cell viability study of primary rat hepatocytes in PNA-10 microcapsule under different culture conditions. (**a**) AOPI stained fluorescent cells. Green and red indicates live and dead cells, respectively. Scale bar: 200 µm. (**b**) Cell viability test. Data represent the mean ± S. D (n = 3). Tukey test, *p < 0.05, **p < 0.01, ***p < 0.001.

Figure 8 represents 3D cultivation of primary hepatocytes (culture conditions: dynamic vs. static) in PNA-10 microcapsules. Fluorescent images show qualitative cell viability where cells were stained with AOPI dye, Fig. 8A. Green and red indicates live and dead cells, respectively. MTT viability was normalized to that of control microcapsule on day 1. Both static and dynamic cell culture condition, cell viability was decreased significantly at day 5 compared to day 1. However, the train of dramatic dcreasing of cell viability over the culture days was found in static condition. Our results indicate that the cell viability of primary rat hepatocytes in PNA-10 microcapsule favor dynamic culture conditions.

## Discussion

In this study, we report a novel strategy to design PNA hydrogel microcapsules using NIM technology and one step method of HepG2 cell encapsulation. Alginate hydrogel microcapsules (AHM) meet several requirements for cell encapsulation. However, one major limitation of AHM is the lack of enough physical and chemical cues at the core of microsphere that impacts the long-term cell growth and development^39,61^. With the aim to enhance physio-chemical properties of AHM for long term cell culture and to build a strategy towards future clinical translations of AHM, we synthesized PNA microcapsule platforms. Prior to PNA microcapsule preparation, PLGA nanofibre mesh was prepared using electrospinning technique. Polymer concentration and types of solvents used to dissolve PLGA are known major game-changing parameters to control the morphology of electrospun fibre^62^. We optimized 20 wt.% PLGA solution (prepared in Chloroform: DMF; 80:20 as solvent) to get a uniform and defect-free nanofibre mesh (Fig. 2A). An air-dried PLGA mesh was used to obtain CPN particles by using grinding mechanism of chattering, chipping, and erosion of material with applied shear forces under liquid nitrogen. Material hardness and temperature of the grinding condition determine the quality of cryo-grinding particles^63,64^. SEM analysis of the CPN particles indicates that the cryogrinding process was successful to convert electrospun nanofibre mesh into intact short fibrous particles at the size of 10–60 µm (Fig. 2B).

Next, the CPN particles were loaded into alginate solution to make PNA microcapsules (400 to 600 µm) with spherical morphology. In this stage, our main aim was to investigate suitability of electrospray technique as a method for preparing ionotropically crosslinked alginate microcapsules containing CPN particles under the conditions that are compatible for cell encapsulation^65–68^. Our material fabrication method indicates that the CPN particles were precisely encapsulated within the PNA microcapsules without any loss of spherical surface morphology up to 28 days of incubation in the cell culture media. This result confirms that PNA microcapsules have good mechanical stability in the cell culture media. The mechanical stability of microcapsules is a major concern in the design of cell immobilization for a therapeutic delivery device, where microcapsule stability is needed to prolong *in vivo* functions^69,70^. Efforts have been given into increasing the mechanical strength of AHM either by adding multilayers of oppositely charged polymer coating^71,72^ or by covalently crosslinking with chemical agents such as glutaraldehyde^73,74^. Both coating or covalent crosslinking techniques require either multi-step process or introduce toxic crosslinking agents into the microcapsules (e.g. glutaraldehyde) which can complicate the encapsulation process. Therefore, the present method of PNA microcapsules preparation provides a simple and straight forward approach to enhance the mechanical strength of microcapsules compared to previously reported approaches.

FTIR results suggest that the addition of CPN in PNA microcapsules does not significantly alter the chemical structure of AHM. The PNA microcapsules containing CPN (10, 30 and 50%) showed greater mass loss and degradation behavior compared to the PNA microcapsules with no CPN i.e. PNA-0 microcapsules indicating that the presence of CPN in AHM play a significant role in altering the thermal degradation pattern of the PNA microcapsules according to loaded concentration^75^. Similarly, the plot from TGA showed the difference in mass loss and thermal stability of the PNA microcapsules (Fig. S2).

The next objective of this study was to encapsulate the HepG2 cells into PNA microcapsules by optimizing electrostatic encapsulation conditions. The working hypothesis was to test whether the PNA provides additional ECM mimicking microstructural and mechanical supports inside the microcapsules. We expected that CPN particles being short nanofibres and spheres being in 3D environments will have the ability to provide cellular contact guidance to facilitate cell growth and development. Although the optimum size of AHM for cell microencapsulation is still debatable, microcapsule of 500 µm diameter has been found to be a good compromise in many studies^66,76,77^. Therefore, in the present study, we optimized our electrostatic encapsulation conditions, mainly flow rate, voltage, distance from tip to the gelling solution, and concentration of CaCl_2_, to produce smooth microcapsules with average diameter 500 µm (Fig. 4). Production of highly controlled monodisperse 3D PNA/HepG2 microcapsules is needed, as uniform cell-encapsulates offer more consistent properties, such as mechanical strength, diffusion and transport of oxygen and nutrients to the core of encapsulated cells.

The viability of HepG2 cells at 24 hours post-encapsulation was more than 97% (Fig. 6A). This indicates that CPN particles did not have short-term detrimental effects on HepG2 cells. However, the viability was dropped down to 80% for PNA microcapsules by day 14 but with considerable differences among all PNA microcapsules. The higher cell viability in PNA-10 microcapsules in an even longer period of culture (Fig. 6B,C) indicates that CPN can contribute to cell adhesion and proliferation under 3D environment. A decrease cell proliferation rate in PNA-30 and PNA-50 microcapsules could be due to the decrease in porosity and poor mass transfer which ultimately affected the permeability of the hydrogel^60^. To confirm the liver-specific metabolic functionality of the 3D PNA/HepG2 microcapsules, urea production, and cytochrome P450 (CYP450) activity assay was performed (Fig. 7). Urea production is an important physiological function of liver cells through active detoxification of ammonia through the urea cycle^78^. CYP450 enzyme activity is a complex phenomenon mediated by activation of nuclear receptors and gene transcription. The enzyme activity is necessary for the detoxification of foreign chemicals and metabolism of drugs. CYP1A1 is one of the major CYP450s involved in drug metabolism^79–81^. CYP1A1, inducible in liver as well as extrahepatic tissue, shows high catalytic activity toward environmental chemicals^82^. In the present study, we also studied the activity of CYP1A1 enzyme and examined significantly higher activity in 3D PNA-10/HepG2 microcapsule compared to control (3D PNA-0/HepG2) at day 14, suggesting that the 3D PNA-10/HepG2 encapsulates may provide a superior conducting environment where HepG2 cells undergo structural and metabolic differentiation. Since our experiments are targeted towards understanding the response of the encapsulated cells, understanding the induction CYP1A1 has been shown to provide important information to predict drug interaction^83^. Further investigation is required to determine whether the 3D PNA-10/HepG2 microcapsule improve liver-specific metabolic functionality. So far, many promising systems for the 3D cultivation of primary hepatocytes have been developed^84,85^. NIM system that we proposed in our current system is conceptually attractive, as NIM mimics many key properties of natural ECMs and provide adequate microenvironments for embedding cells under real 3D conditions.

In summary, we designed and fabricated alginate-based 3D microcapsules by incorporating cryo-fractured electrospun nanofibres of PLGA in different weight percentages. The incorporation of nanofibres caused no change in the chemical structure of the alginate hydrogel microcapsule and its spherical morphology for several weeks in a cell culture media. We optimized our electrostatic encapsulation conditions to produce 3D microcapsules of HepG2 with an average diameter of 500 µm. Our finding suggests that the 3D PNA-10/HepG2 microcapsule improved overall suitability for cell proliferation, toxicity, and metabolic activity compared to PNA-0/HepG2 microcapsule as a control. These results provide a baseline information to improve the material’s design and configurations for optimal *in vitro* cell growth and development. Factors including culture conditions (dynamic vs. static) also have an important influence on the 3D cultivation of primary hepatocytes (Fig. 8). Development of this 3D cell microcapsules by incorporating co-cultures of other hepatic cells, such as primary hepatocytes, kupffer cells, stellate cells, etc., may further improve characteristics, and prediction of human liver-related functionalities in *in vitro* 3D system. However, several additional assessments are still needed to validate and develop 3D PNA-10 microcapsule into a fully functional *in vivo* mimicking architecture of liver which has potential use for preclinical study, toxicology, and pharmacological drug screening.

## Methods

### Electrospinning of PLGA nanofibre

PLGA (75:25, inherent viscosity 0.55–0.75 dL/g), was purchased from Durect Corporation (Birmingham, AL, USA). The PLGA solutions of 15, 20 and 25% (w/v), was prepared in the mixture of chloroform and DMF (80:20). Electrospun nanofibre mesh of PLGA was prepared as described in previous publications^86,87^ with some modification. In brief, the electrospinning apparatus consisted of a syringe pump, a high-voltage power supply, and a BD Luer-Lok syringe with an attached 22 G-diameter hypodermic needle. The needle-to collector distance was maintained at 75 mm, with an applied voltage of 20 kV. The feeding rate of the solution was precisely controlled by a syringe pump system, which was adjusted to a flow rate of 4 mL/h. The fibres deposited onto an aluminum foil-wrapped rotating collector were left overnight in dust-free conditions at room temperature, to allow complete solvent evaporation. The nanofibre meshes were removed from the collector and detached from the aluminum foil for further characterization.

### Production of cryoground PLGA nanofibre (CPN)

Electrospun nanofibre mesh of PLGA was subjected through the cryogrinding process as previously described^64^ with some modification to the obtained ground powder of nanofibres. A cryogenic impact grinder (6770 Freezer Mill, Spex, USA) with a self-contained liquid nitrogen bath (4–5 L) was used. About 0.2 g of PLGA nanofibre mesh was cut into the small pieces of unit cm^2^ size and put into a 25 ml polycarbonate grinding vials for pulverization. After a pre-cooling period (5 min), six working cycles were used for each grinding. Each cycle consisted of grinding and re-cooling periods (1 min). The applied impact bar frequency was 14 Hz. The ground fibre was dispersed in ethanol and filtered through sterile 70 µm sieve for the smaller and uniform size of CPN. Dry powder of CPN was collected after complete evaporation of ethanol under the hood.

### Surface morphology analysis

The surface morphology of electrospun PLGA nanofibre mesh and CPN was analyzed using a scanning electron microscope (SEM, Hitachi SU8000, Tokyo, Japan). Small cut pieces of mesh and CPN samples were deposited on copper tape and sputter-coated with gold using a Polaron SEM coating system (Quorum Technologies, East Sussex, UK) for 90 seconds at 15 mA. SEM images were taken at an accelerating voltage of 10 kV and 10 μA current. Image-Pro Plus 6.0 software (Media Cybernetics, Inc., Rockville, MD, USA) was used to measure nanofibre diameters in SEM images.

### Fabrication of PNA microcapsules

Ultra-pure alginate (PRONOVA UP LVG) was purchased from Novamatrix (Industriveien 33 N-1337 Sandvika Norway). 2% (w/v) alginate solution was prepared in 1X HBSS buffer (Life Technology, Gaithersburg, MD, USA). CPN was mixed with an alginate solution in different weight percent of the dry weight of alginate. CPN was first dispersed in DI water under sonication and then combined with alginate solution. For better dispersion of CPN, the mixture solution was pipetted and vortexed for 5 min. PNA microcapsules were obtained by electro-spraying the dispersed solution of CPN-alginate using a previously published method^65,66^. In brief, the CPN-alginate solution was drawn into a syringe fitted with a 24-gauge angiocatheter, pierced at the hub with a 23-gauge needle to serve as the positive electrode in the electrostatic encapsulation process. The syringe was loaded onto a syringe pump and arranged in such a way that the droplets ejected from the angiocatheter would fall orthogonally onto the surface of 150-mM calcium chloride (CaCl_2_) solution. The distance from the angiocatheter tip to the surface of the CaCl_2_ was fixed at 25 mm with an applied voltage of 5.5 kV. Pump flow rates were set at 18 mL/min and the ground electrode was immersed in the CaCl_2_ receiving bath. After fabrication, PNA microcapsules were washed twice with DI water before subjected for further characterization. The control microcapsules without CPN were also fabricated using the alginate solution only.

### Morphology and stability study

Morphology of the fabricated PNA microcapsules was studied by using an optical microscope (EVOS® XL Core Imaging System) and SEM. Optical images were taken while microcapsules were in the cell culture condition. SEM images were taken after PNA microcapsules were lyophilized utilizing the same procedure stated previously. PNA microcapsules were cryo-fractured in liquid nitrogen and lyophilized to observe their internal morphology using SEM. Size distributions of the microcapsule were measured from optical images using Image J software. Stability of the PNA microcapsules was studied by incubating them into 24 well plates with complete DMEM culture media in the rotator at 50 rpm speed for four weeks.

### FTIR and TGA analysis

Fourier-Transform Infrared Spectroscopy (FTIR) was used to identify functional groups and chemical interactions between alginate and CPN in the 3D PNA. FTIR spectra were obtained with a Varian 670 FT-IR Spectrophotometer (Varian, Inc., Palo Alto, CA, USA) in the range of 4000 to 600 cm^−1^ region.

Thermogravimetric Analysis (TGA) was performed to study the mass change and thermal behavior of fabricated microcapsule. TGA thermograms were obtained with a TA Instrument Q5000 (TA Instrument, New Castle, DE, USA) at heating rates of 10 °C/min from 25 to 700°C under nitrogen.

### HepG2 cell culture

HepG2 cells (human liver carcinoma cell line, ATCC® HB-8065™) (ATCC, Manassas, VA, USA) were maintained in standard Dulbecco’s Modified Eagle’s Medium (DMEM) supplemented with 10% fetal bovine serum (Invitrogen, Waltham, MA, USA), 1% Pen strip (100×) (Life Technology, Gaithersburg, MD,USA) and 0.12% insulin (Life Technology, Gaithersburg, MD,USA). The cells were grown in 75 cm^2^ tissue culture flasks at 37 °C in a 5% CO_2_ humidified environment. At confluence, cells were trypsinized with 0.25% Trypsin/EDTA (Invitrogen, Waltham, MA, USA), pelleted by centrifugation and finally resuspended with fresh medium to the desired cell density.

### HepG2 cell encapsulation in PNA microcapsules (3D PNA/HepG2)

HepG2 cells at a density of 1.5 × 10^7^ cells/mL were suspended in a 2% sodium alginate solution (with or without CPN) at 1:1 ratio. The resulted mixture of 1% alginate solution with the HepG2 cells was electrosprayed under the same condition as described in earlier fabrication of PNA microcapsules section. The encapsulated cells in PNA microcapsules (3D PNA/HepG2) were quickly transferred into 1X HBSS buffer for about 30 min and then transferred into 24 well plates with complete DMEM media for further culture. The 3D PNA/HepG2 were cultured under the same condition used for HepG2 cells. Culture media was changed and replenished with fresh warm (37 °C) DMEM media at each time points per experimental setting. The changed media was collected and stored at −20 °C for analysis. Morphology and size distribution were also analysed as described method in earlier section.

### Primary hepatocyte encapsulation in PNA microcapsules (3D PNA/PH)

Method for encapsulation of primary hepatocytes (PH) in PNA microcapsules was exactly the same as HepG2 cells. Briefly, freshly isolated primary rat (Wistar) hepatocytes were purchased from the Triangle Research Laboratory (Research Triangle Park, NC). Cell counting and viability measurement of fresh hepatocytes obtained from the company was performed by trypan blue (TB) assay before these cells were assigned to a designated experimental purpose and processed accordingly. Cells were used for encapsulation within 6–8 hours of their isolation. After encapsulation as previously described, microcapsules were cultured in static and dynamic (3D rotator at 40 rpm speed) condition. The viability of encapsulated hepatocytes was evaluated by measuring the reduction of tetrazolium salt [3-(4, 5-dimethylthiazol-2-yl)–2–5 diphenyltetrazolium bromide; MTT as previously reported^88^ using commercially available Vybrant™ MTT Cell Proliferation Assay Kit (Thermo Scientific, catalog No: V13154, Waltham, MA, USA) according to company protocol. The viability was presented as the percentage of viable cells in different time points relative to the viability of control.

### HepG2 cell viability and attachment

Cell viability was monitored with Trypan Blue (TB) assay after retrieving the encapsulated cells. The cultured PNA/HepG2 microcapsules were quickly rinsed with 1X DBPS (Life Technology, Gaithersburg, MD, USA) twice and de-gelled by using sodium citrate (100 mM) solution. The resulted solution was centrifuged to retrieve the cell pellet which was resuspended in fresh media after decanting the supernatant. The retrieved suspension was stained with TB reagent and cell viability was calculated after counting the live or dead cells using haemocytometer.

Cell attachment and distribution within the microcapsule were studied by SEM analysis. The samples were prepared by rinsing the 3D PNA/HepG2 with DPBS (2 brief rinses) followed by fixation with 4% glutaraldehyde (Sigma Aldrich, St. Louis, MO, USA) for 30 min at 4 °C. After fixation, samples were briefly rinsed with deionized (DI) water (2 times) and dehydrated (sequential incubations in 30, 50, 75 and 100% ethanol, 10 mins each) at room temperature. The samples were left to dry in a sterile fume hood for 24 h before SEM imaging.

### Fluorescence imaging and analysis

Fluorescence imaging of 3D PNA/HepG2 was performed by staining with acridine orange and propidium iodide (AOPI) dye (Nexcelom Bioscience, Lawrence, MA). At different time points, cultured media was aspirated from the wells, and microcapsules were washed with DPBS twice to remove FBS. Then, stained with 15 µl dye and incubated at 37 °C for 10 min. Z-stack fluorescence images were photographed under an Olympus IX83 microscope using Olympus cell Sens Dimension software (Olympus Corporation, Shinjuku, Tokyo, Japan).

### Lactase dehydrogenase (LDH) assay

LDH was quantified in collected media at different time periods with a Pierce LDH cytotoxicity assay kit (Thermo Scientific, CatLog No: 88953, Waltham, MA, USA). Briefly, 50 µL of each collected sample medium was transferred to a 96-well flat-bottom plate in triplicate wells along with LDH positive control (as mentioned in the kit) and blank media as a negative control. Then, 50 µL of the reaction mixture was added in each well, and the plate was incubated at room temperature for 30 minutes at dark condition. The reaction was stopped by adding 50 µL of Stop Solution to each sample wells and mixed by gentle tapping. The absorbance of the assay solution was measured on a microplate reader (BioTek Inc., Winooski, VT, USA) at 490 nm and 680 nm wavelength to calculate the cytotoxicity.

### Urea and CYP450 assay

Urea assay was performed after stimulating the microcapsules with 5 mM NH_4_Cl for 24 h as described previously^88^ with some modification. And, commercially available urea assay kit (BioChain, Newark, CA, USA, catalog No: Z5030016) was used to calculate the urea production in collected cultured media according to the manufacturer’s instruction. Briefly, 50 µL of water (blank), standard (as provided in the kit) and samples were taken in triplicate into separate wells of 96 well plates. Then, 200 µL working reagent was added and incubated 50 min at room temperature. Optical density was read at 430 nm using microplate reader and urea concentration (µg/ml) in the collected sample was calculated.

CYP1A1 enzyme activities were measured by P450-Glo™ CYP1A1 assay kit (Promega Co., Madison, WI, USA, catalog No: V8751) as described previously^89^ with some modification. Briefly, CYP1A1 activity was induced by incubating microcapsules in media supplemented with 300 μM omeprazole. Cultured media alone was used as a control. All microcapsules were incubated with complete media supplemented with 100 μM Luciferin-CEE for 5 h. An aliquot (25 μl) of the medium was transferred to 96-well opaque white luminometer, and luciferin detection reagent (25 µl) was added to each well. After sitting the samples at room temperature for 20 min, luminescence was measured using microplate reader.

### Statistical analysis

All results were expressed as mean ± S.D. Data were analysed for significance with OriginPro software (Origin Lab, Northampton, MA, USA) using a one-way analysis of variance (ANOVA). Post hoc Tukey’s test was performed with ANOVA for multiple comparisons. The α-value was set to 0.05 and p-values less than 0.05 were considered statistically significant.

## Supplementary information

Nano-fibre Integrated Microcapsules: A Nano-in-Micro Platform for 3D Cell Culture

## Data Availability

The datasets generated during and/or analysed during the current study are available from the corresponding author upon request.
