# Author Correction: Nano-fibre Integrated Microcapsules: A Nano-in-Micro Platform for 3D Cell Culture

**DOI:** 10.1038/s41598-023-28208-9

**Published:** 2023-01-23

**Authors:** Shalil Khanal, Shanta R. Bhattarai, Jagannathan Sankar, Ramji K. Bhandari, Jeffrey M. Macdonald, Narayan Bhattarai

**Affiliations:** 1 Department of Applied Science and Technology, North Carolina A&T State University, Greensboro, NC USA; 2 Department of Chemistry, North Carolina A&T State University, Greensboro, NC USA; 3 Department of Biology, North Carolina A&T State University, Greensboro, NC USA; 4 Department of Biology, University of North Carolina Greensboro, Greensboro, NC USA; 5 Department of Mechanical Engineering, North Carolina A&T State University, Greensboro, NC USA; 6 Department of Biomedical Engineering, University of North Carolina, Chapel Hill, NC USA; 7 Department of Chemical, Biological, and Bioengineering, North Carolina A&T State University, Greensboro, NC USA

Correction to: *Scientific Reports* https://doi.org/10.1038/s41598-019-50380-0, published online 27 September 2019

This article contains an error in Figure 5, where panel ‘PNA-30/HepG2 Day 7’ is a duplication of panel ‘PNA-0/HepG2 Day 14’, and ‘PNA-10/HepG2 Day 14’ is a duplication of ‘PNA-50/HepG2 Day 1’.

The corrected Figure 5 and its accompanying legend appear below:

**Figure 5:**
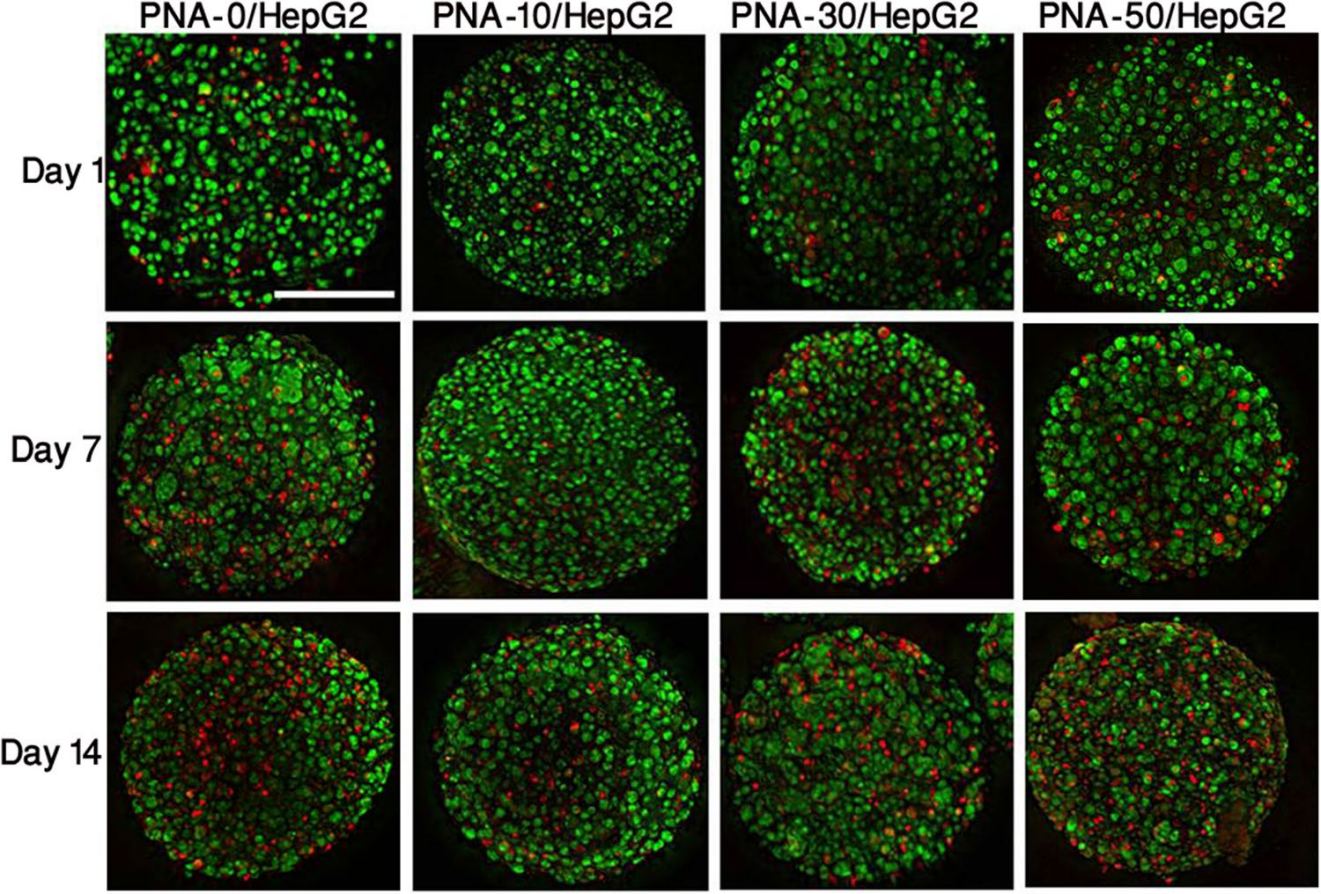
Fluorescent images of dye-labeled 3D PNA/HepG2 microcapsules at different time points. Green and red colour indicates live and dead cells, respectively, and were uniformly distributed in all compositions of the PNA microcapsules. Scale bar = 200 µm.

