# Supplementary material for: Nano-fibre Integrated Microcapsules: A Nano-in-Micro Platform for 3D Cell Culture
Source: Sci Rep. 2019 Sep 27;9:13951. doi: 10.1038/s41598-019-50380-0 (PMC6765003; doi:10.1038/s41598-019-50380-0)
Supplement: Supplementary file 1 — Nano-fibre Integrated Microcapsules: A Nano-in-Micro Platform for 3D Cell Culture [file 41598_2019_50380_MOESM1_ESM.docx]

Supplementary information

**Nano-fibre Integrated Microcapsules: A Nano-in-Micro Platform for 3D Cell Culture**

Shalil Khanal ^1^, Shanta R. Bhattarai ^2,4,7^, Jagannathan Sankar ^3^, Ramji Bhandari ^4^, Jeffrey Macdonald^5^, and Narayan Bhattarai ^6, *^

^1^Department of Applied Science and Technology, North Carolina A&T State University, Greensboro, NC, USA

^2^Department of Chemistry, North Carolina A&T State University, Greensboro, NC, USA

^3^Department of Mechanical Engineering, North Carolina A&T State University, Greensboro, NC, USA

^4^Department of Biology, University of North Carolina, Greensboro, NC, USA

^5^Department of Biomedical Engineering, University of North Carolina, Chapel Hill, NC, USA

^6^Department of Chemical, Biological, and Bioengineering, North Carolina A&T State University, Greensboro, NC, USA

^7^Department of Biology, North Carolina A&T State University, Greensboro, NC, USA

*Author to whom correspondence should be addressed


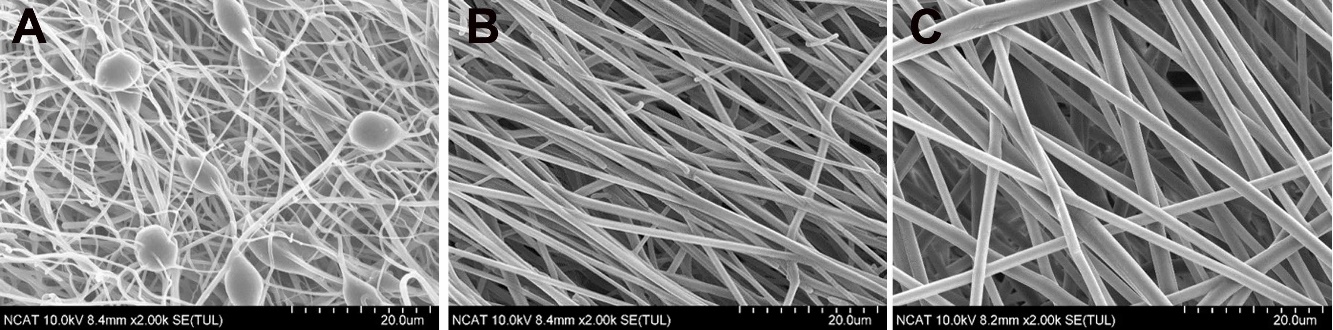


Figure S1: SEM image of electrospun PLGA nanofibre mesh prepared by using different concentrations. (A) 15%, (B) 20%, (C) 25%


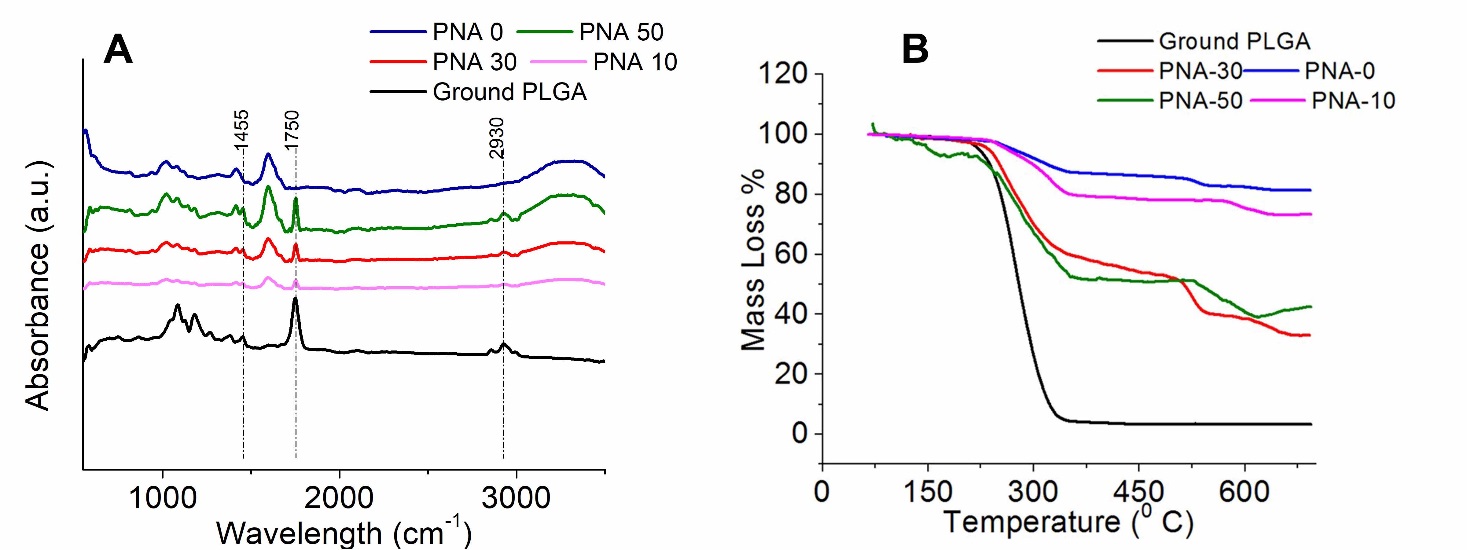


Figure S2: (A) FTIR spectra of ground PLGA fibre and four different types of PNA microcapsule. Typical absorption bands for PLGA are located at: 3200-3500 cm^-1^ for OH stretching, 2850-3000 cm^-1^ for -CH stretching, 1700-1850 cm^-1^ for carbonyl carbon –C = O stretching, 1050-1250 cm^-1^ for C–O stretching and 1390-1460 cm^−1^ for bending of -CH [^1^](#_ENREF_1). Fiber-loaded microcapsule showed all the peaks found in ground PLGA and no changes in the position of these peaks rather than showing the absorption peaks as a result of the simple superposition of their separated components in the infrared spectra. These results suggest that the addition of fibre did not significantly alter the chemical structure of alginate hydrogel.

(B) TGA thermograms of ground PLGA fibre and four different types of PNA microcapsule. Ground PLGA showed its thermal stability up to 240 ºC. Almost 100% weight loss occurred in the range of 240-365 °C which indicates thermal decomposition and evaporation of the material [^2^](#_ENREF_2). The curve of alginate only microcapsule (PNA-0) shows a weight loss in two distinct stages. The first stage between 30 and 220 ^0^ C shows about 18% loss in weight which corresponds to the loss of adsorbed and bound water. The second stage of weight loss starts from 235 to 325 ^0^C, during which there was a 58% weight loss due to the degradation of alginate [^3^](#_ENREF_3). The other microcapsule containing ground PLGA shows greater mass loss and degradation behavior than F0 which indicates that the presence of ground PLGA in alginate hydrogel plays a significant role in altering the degradation pattern of the microcapsule according to loaded concentration [^4^](#_ENREF_4).


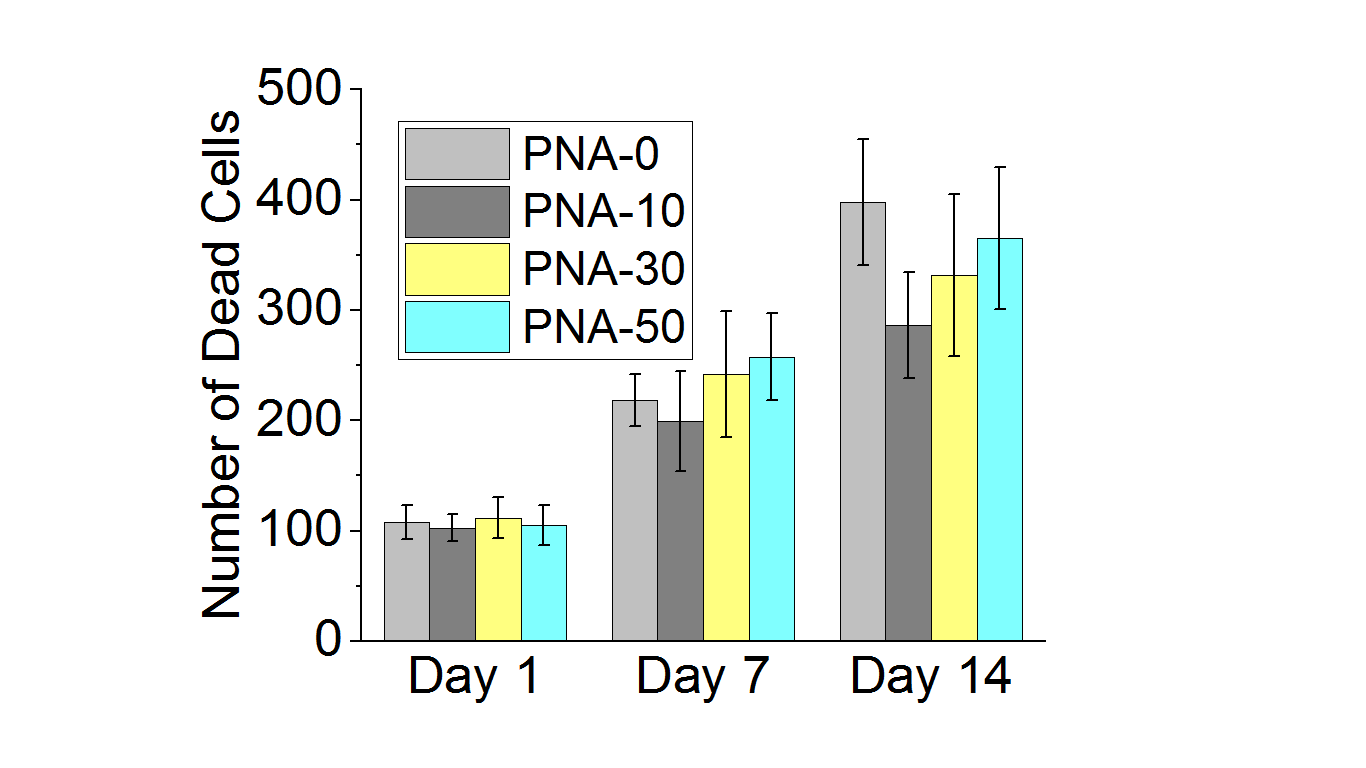


Figure S3: Number of dead cells count per microcapsules at each time point


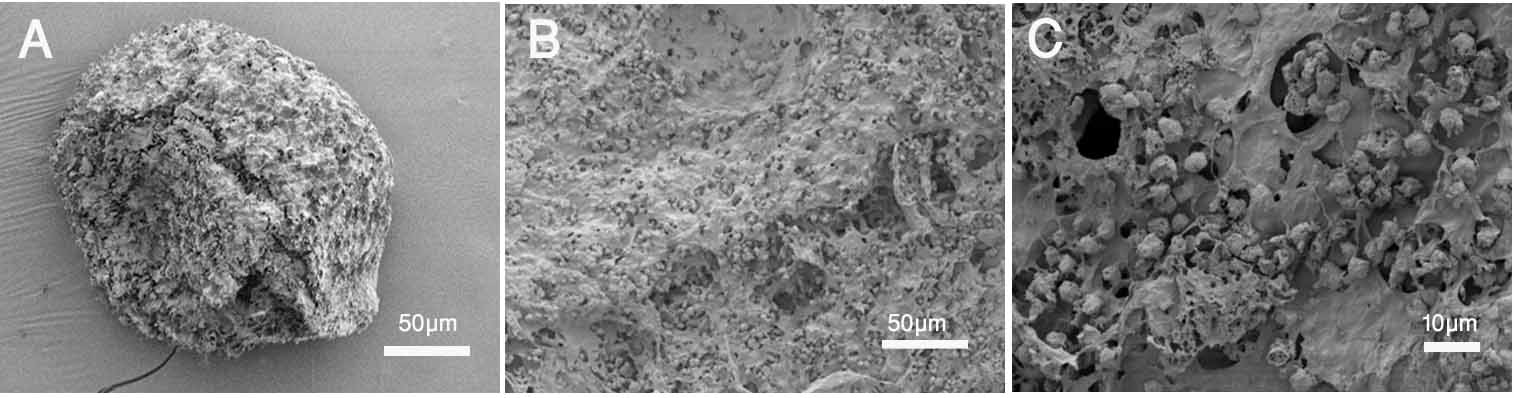


Figure S4: SEM image of 3D PNA-10/HepG2: (A) single dry microsphere, (B and C) cross section of microcapsule showing the distribution of encapsulated cells.


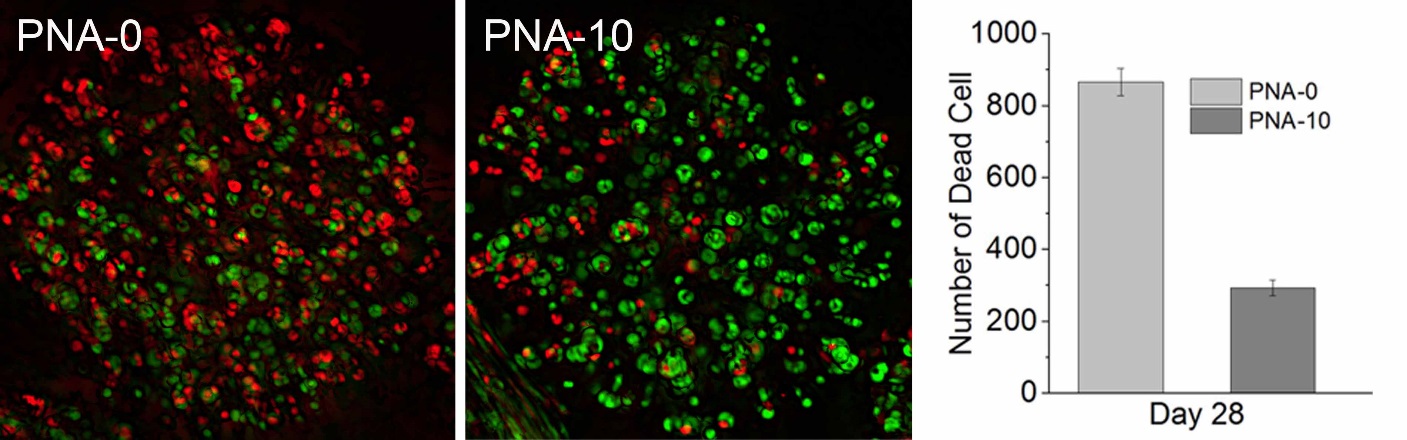


Figure S5: Fluorescent Images of dye-labeled 3D PNA/HepG2 microcapsules at day 28. Green and red indicate live and dead cells, respectively. The graph represents comparison of number of dead cells in the corresponding microcapsules*.*

1 Pirooznia, N., Hasannia, S., Lotfi, A. S. & Ghanei, M. Encapsulation of alpha-1 antitrypsin in PLGA nanoparticles: in vitro characterization as an effective aerosol formulation in pulmonary diseases. *Journal of nanobiotechnology* **10**, 20, doi:10.1186/1477-3155-10-20 (2012).

2 Khalil, K. A., Fouad, H., Elsarnagawy, T. & Almajhdi, F. N. Preparation and characterization of electrospun PLGA/silver composite nanofibers for biomedical applications. *Int J Electrochem Sci* **8**, 3483-3493 (2013).

3 Sadeghi, M., Shafiei, F., Mohammadinasab, E., Mansouri, L. & Shasavar, H. *Synthesis of Biodegradable Hydrogel Based on H-alginate-g-poly(AMPS)*. Vol. 11 (2014).

4 Kumar, T. M. M. & Praveen, D. Impedance analysis of Sodium alginate : Graphene oxide composite. *IOP Conference Series: Materials Science and Engineering* **310**, 012150 (2018).
